# Post-exercise Hypotension in Patients With Coronary Artery Disease

**DOI:** 10.3389/fphys.2021.788591

**Published:** 2021-12-22

**Authors:** Ferdinando Iellamo, Marco Alfonso Perrone, Giuseppe Caminiti, Maurizio Volterrani, Jacopo M. Legramante

**Affiliations:** ^1^Dipartimento di Scienze Cliniche e Medicina Traslazionale, Università Tor Vergata, Rome, Italy; ^2^Istituto di Ricovero e Cura a Carattere Scientifico San Raffaele Pisana, Rome, Italy; ^3^Dipartimento di Medicina dei Sistemi, Università Tor Vergata, Rome, Italy

**Keywords:** exercise, hypotension, baroreflex, neural regulation, hemodynamics, ischemic heart disease, post-exercise hypotension

## Abstract

**Background:** Blood pressure (BP) and hemodynamic changes occurring in the recovery phase after a single bout of exercise have not been extensively studied in coronary artery patients, despite the potential clinical implications of reducing BP through exercise. This study aimed at investigating the hemodynamic and arterial baroreflex mechanisms possibly involved in post-exercise hypotension (PEH) in patients with coronary artery disease.

**Methods**: In 42 normotensive coronary artery patients undergone a Cardiac Rehabilitation Program, we evaluated before and after their daily exercise training session: blood pressure (BP) and heart rate (HR). In a subgroup (*n* = 29), daily BP profile was also evaluated by ambulatory BP monitoring. In those patients showing PEH (*n* = 15), we evaluated: Cardiac Output (CO), Stroke Volume (SV), total peripheral resistances (TPR), forearm (FVR) and calf (CVR) vascular resistances, and spontaneous baroreflex sensitivity (BRS).

**Results:** After exercise TPR was significantly reduced with a similar contribution from CVR and FVR, whereas CO and SV significantly increased. BRS showed a significant reduction mainly due to a BRS decrease in response to hypertensive stimuli. Systolic BP (SBP) was significantly reduced for 12 h after the end of a single exercise session.

**Conclusion:** These findings indicate that in coronary artery patients, the recovery phase after exercise is characterized by PEH which is mediated mainly by a generalized peripheral vasodilation and appears to influence BP behavior throughout the daily life. Finally, the cardiac component of the arterial baroreflex seems to contribute indirectly to BP reduction occurring after exercise.

## Introduction

It has been widely reported that after a single out of dynamic exercise, blood pressure (BP) is usually reduced below pre-exercise control value thus configuring the phenomenon indicated as post-exercise hypotension (PEH; Kenney and Seals, 1993). We reported that in hypertensives, PEH is mediated by a peripheral vasodilation, which may involve metabolic factors linked to post-exercise hyperemia, overriding a concomitant, reflex sympathetic activation directed to the vasculature, with the possible aim to oppose excessive decreases in BP (Legramante et al., 2002). In addition, we have suggested that baroreflex mechanisms controlling heart period retain the potential for a greater opposition to hypotensive stimuli (Legramante et al., 2002), thus contributing to buffer excessive BP decreases.

Because exercise training has been shown to produce beneficial effects in many cardiovascular diseases (Fletcher et al., 1996) and to improve prognosis after myocardial infarction (O’Connor et al., 1989) nowadays, regular physical activity is recommended as a major strategy in the secondary (in addition to primary) prevention of cardiovascular disease. Thus BP reduction may be an important target of secondary prevention even in normotensive patients with established coronary artery disease (CAD), since even small reductions in BP might be useful with regard to coronary risk (Oparil, 1999).

To our knowledge, however, only one small study investigated changes in BP occurring in the recovery phase after exercise in CAD patients (Fagard and Vanhees, 2000), with no clear-cut results. Little is known as the duration of PEH, because BP monitoring was discontinued shortly after exercise and even less as the persistence of BP decrease.

Ambulatory blood pressure monitoring (ABPM) provides information about the dynamics of BP, (and heart rate as well) during daily life, and is now being increasingly recommended for both diagnostic and therapeutic purposes (Mallion and Baguet, 2006).

Accordingly, the aim of the present study was to investigate BP, hemodynamic, and autonomic (baroreflex) changes occurring after an exercise training session in CAD patients enrolled in a supervised exercise training program and whether BP lowering after a single exercise session is sustained over time while patients have continued their daily activity.

## Materials and Methods

### Subjects

The study included 42 normotensive CAD male patients (mean age 59 ± 7.2) consecutively referred to our Cardiac Rehabilitation Center under reimbursement of the Italian Health Care system. Criteria for eligibility were male sex, sinus rhythm, and normal BP values (≤ 130/80 mmHg) Exclusion criteria were hypertension, congestive heart failure, residual angina, insulin-treated diabetes, major arrhythmias, age > 70 years, EF < 45%. Diet and medications were not altered during the study. The study was performed during a residential cardiac rehabilitation program. Informed consent was obtained from each subject, and the study was approved by the Ethical Committee of the Cardiac Rehabilitation Center.

### Measurements

#### Blood Pressure and Heart Rate

In all patients, BP and HR were measured in the sitting posture, with a conventional sphygmomanometer, before and after their daily exercise training session. In a subgroup of patients (*n* = 29), a 24-h non-invasive ambulatory BP monitoring (ABPM; Spacelabs, United States) was performed twice. In order to study the baroreflex control of sinus node, in a subgroup of subjects showing PEH (*n* = 15), BP was continuously and non-invasively measured by Finapres (Finapres, Ohmeda 2,350, Englewood, CO, United States). The output of the Finapres was transmitted *via* the RS 232 serial port and processed by a software program written in our laboratory. Baroreflex sensitivity (BRS) was assessed by means of the sequences technique (Iellamo et al., 1994).

#### Systemic Hemodynamics

In the same subgroup of patients featuring PEH (*n* = 15), an echocardiographic (Sequoia, Acuson, United States) parasternal long-axis view was used to measure end-diastolic and end-systolic left ventricular diameters as well as the aortic ring diameter. The instantaneous flow velocity in the ascending aorta was measured using continuous-wave Doppler. Stroke volume (SV) was calculated as the product of mean time-velocity integrals and the cross-sectional area of the aortic orifice, as done in previous studies (Ihelen et al., 1984; Iellamo et al., 1994; Legramante et al., 2002). Cardiac output (CO) was calculated as the product of SV and HR. Total peripheral resistance (TPR) was calculated in dynes sec^−1^ cm^−2^ according to the following formula: TPR = 80 mean blood pressure/CO.

#### Regional Hemodynamics

Brachial and femoral artery ultrasound scans were obtained with a 7 MHz linear array transducer (Celermajer et al., 1992). The diameter of the arteries was measured from B-mode images over longitudinal sections. Arterial blood flow velocity was measured from pulsed Doppler signal. At least 5 measurements were averaged. Volumetric flow was calculated for each study by multiplying the angle corrected velocity time integral of the Doppler signal by the heart rate and the vessel cross-sectional area. All measurements used for flow calculations were obtained simultaneously.

#### Experimental Protocol

After the patients had sat quietly for 15 min, BP was measured twice, 5 min apart, and the measurements were averaged. After the instrumentation, following a 20 to 25-min adaptation period to the supine position patients underwent baseline echocardiographic, vascular echo-doppler examination, and continuous BP and HR recordings for 10 min.

After baseline measurements, patients underwent their normal exercise training session on a cycle ergometer according to their Rehabilitation Program, as previously reported (Iellamo et al., 2000). Briefly, the training program consisted of 2 daily sessions of 30 min of stationary cycling 6 times a week for 2 weeks (24 sessions overall) combined with calisthenics. Training intensity was graded according to 85% of the HR max reached in an initial exercise test.

60 min after the end of the exercise, during which patients sat quietly in the laboratory, post-exercise BP was measured by sphygmomanometer as described above.

Thereafter, the first 15 consecutive patients who exhibited PEH repeated the echocardiographic, vascular echo-doppler, and the continuous BP and HR recordings in the supine position as before exercise. BP was measured again 120 min after the end of the exercise by sphygmomanometry.

In patients (*n* = 29) who underwent ABPM, the two recordings were performed in an “exercise” and in a “control day.” The patients were instructed to engage in normal daily activities and to avoid strenuous exercise during the 24-h monitoring. On the “exercise day” ABPM was initiated ~15 min before subjects carried out the regular exercise training sessions. On the “control day,” the patients abstained from physical activity including the exercise training program. The participants were asked to maintain the same daily routines (including time of sleeping) as after their regular exercise training session.

All the studies were performed in the final week of the training program, and ABPMs were performed at least 3 days apart.

#### Spontaneous Baroreflex Analysis

Details of this analysis have been previously described (Iellamo et al., 1994, 1996, 1999, 2001). Briefly, the beat-by-beat time series of SBP and pulse interval (PI) were scanned by a computer to identify sequences of 3 or more consecutive beats in which SBP and PI changed in the same direction (either increasing, i.e., “up-sequences,” or decreasing, i.e., “down-sequences”). A linear regression was applied to each sequence and the mean individual slope of the SBP/PI relationship, obtained by averaging all slopes computed within the test period, was calculated and taken as a measure of the integrated BRS (Bertinieri et al., 1988; Iellamo et al., 1994). We evaluated the occurrence of baroreflex sequences by the engagement time (eng %), which calculates the fractional occurrence of the sequences (Legramante et al., 2001).

#### Statistics

Each variable was checked for normality of distribution by the Kolmogorov-Smirnov test. When normality test passed, paired *t*-test or one-way ANOVA for repeated measures was used, when appropriate, to compare pre- and post-exercise values for each of the reported variables. The Wilcoxon signed rank test or the Friedman ANOVA on ranks for repeated measures were used, when appropriate, for nonnormally distributed variables. Values are expressed as means ± SEM. Differences were considered statistically significant when *P* was <0.05.

## Results

### Exercise Training

The mean workload attained during the exercise training session was 74.3 ± 2.4 Watt (range 70–110 Watt) and the average HR max reached 146 ± 2.9 beats/min (87 ± 2.0% of the maximal age-predicted HR). No patients complained any symptoms during and after the exercise training sessions.

### Blood Pressure

**Figure 1:**
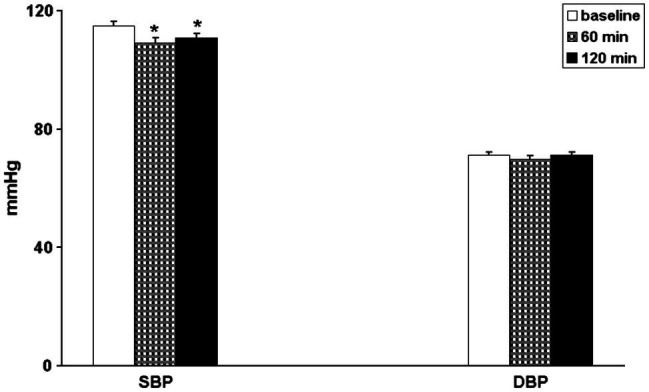
Bar graphs showing systolic (SBP) and diastolic (DBP) blood pressure in 42 coronary artery patients in baseline conditions (**open bars**) and 60 min (**hatched bars**) and 120 min (**black bars**) after exercise. ^*^p < 0.05 vs. baseline.

After exercise BP was lower than in baseline conditions in 76% (32 out of 42) for systolic and in 52% (22 out of 42) for diastolic values of the patients. 60 and 120 min after the end of exercise, SBP still showed a significant decrease, whereas DBP did not showed significant difference as compared to the pre-exercise values (Figure 1). HR did not show significant changes both at 60 and 120 min after exercise (from 69.2 ± 1.2 b/min to 69.1 ± 1.4 b/min and to 68.5 ± 1.3 b/min, *p* = 0.772).

**Figure 2:**
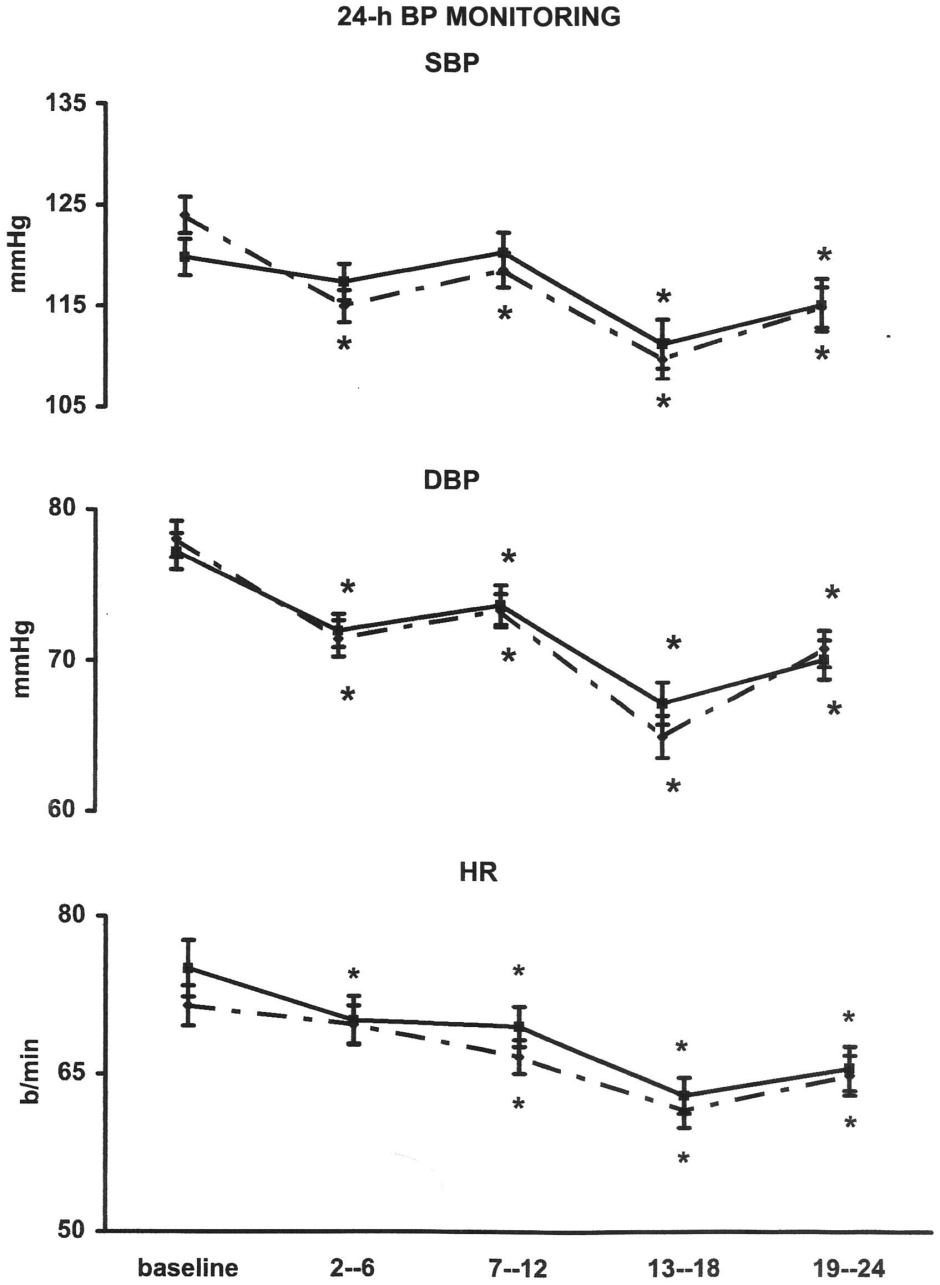
Line graph shows average values for systolic (SBP) and diastolic (DBP) blood pressure and heart rate (HR) in 29 coronary artery patients at 2nd–6th, 7–12th, 13–18th, and 19–24th interval hours after exercise training session (¨) compared with measurements taken at the same time of day on a nonexercise control day (▪). ^*^p < 0.05 vs. baseline.

**Figure 3:**
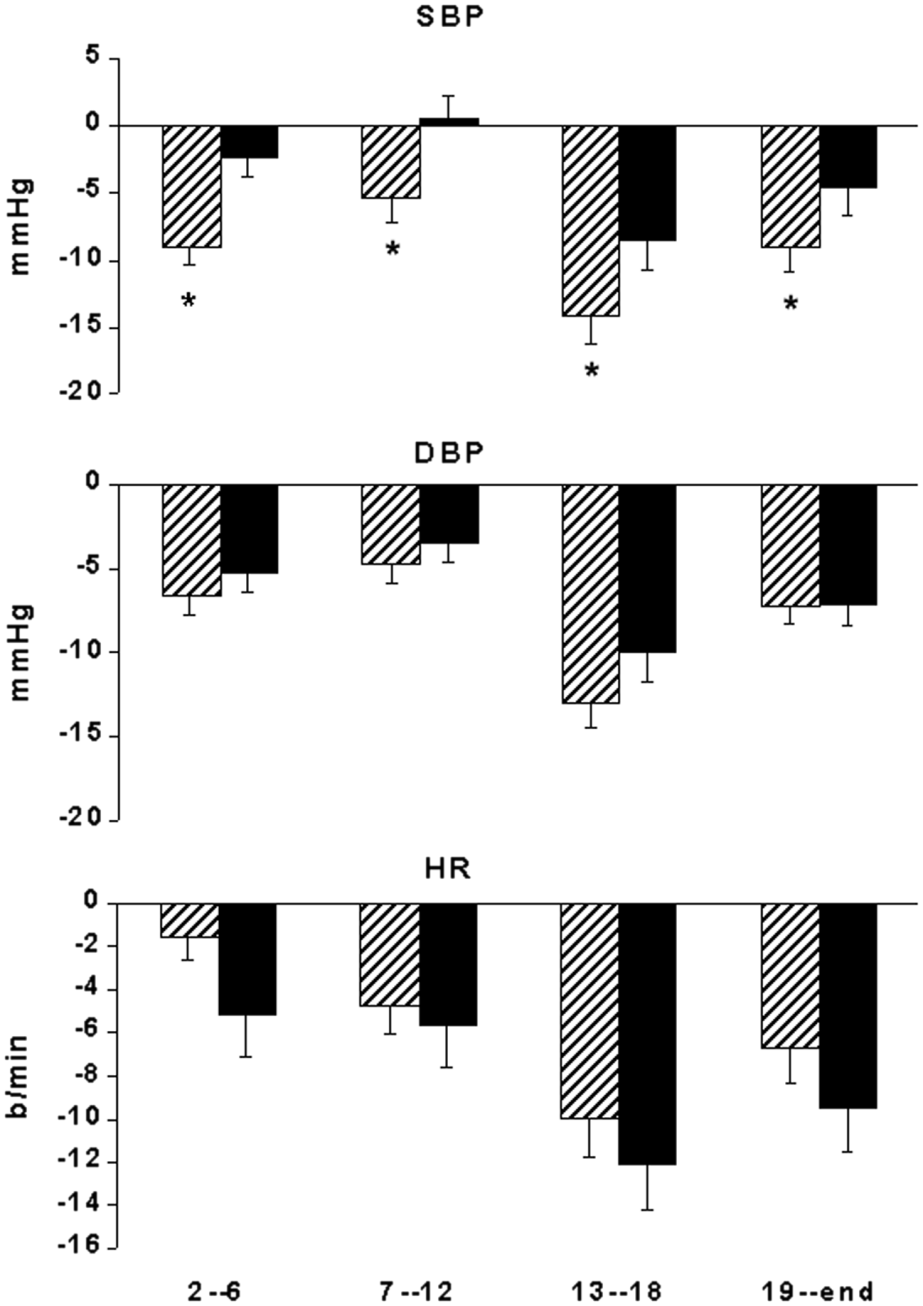
Bar graphs showing systolic (SBP) and diastolic (DBP) blood pressure and heart rate (HR) changes from the start of ABPM in 29 coronary artery patients at 2nd–06th, 7–12th, 13–18th, and 19–24th interval hours after exercise training session (**hatched bars**) compared with measurements taken at the same time of day on a nonexercise control day (**black bars**). ^*^p < 0.05 vs. nonexercise control day.

Baseline BP did not show significant differences between the ABPM of the “control” and the “exercise day” (Figure 2). In the “exercise day,” SBP was significantly reduced as compared to the baseline for 12 h after the end of the exercise, whereas in the “control day,” no significant changes in SBP have been reported during the same time interval (Figure 2). SBP reductions were significantly greater during the “exercise day” as compared to the “control day” for the first 12 h after the end of the exercise training session (Figure 3). DBP and HR showed a similar behavior in the two experimental sessions (Figures 2, 3). During the night (13th-24th hours), as expected, all the cardiovascular variables showed a significant reduction that was independent from the exercise training session (Figure 2), even though SBP reductions were significantly greater during the “exercise day” as compared to the “control day” (Figure 3).

### Systemic and Regional Hemodynamics

In a subgroup of patients exhibiting PEH (*n* = 15), systolic and diastolic BP reduction (Table 1) was accompanied by a significant and substantial decrease in TPR and by a significant increase in SV and in CO. HR was not significantly different before and after exercise (Table 1). The vasodilation appeared generalized including both exercising, calf (CVR), and non-exercising, forearm (FVR), muscular districts (Table 1).

**Table 1:** Systemic and peripheral hemodynamics in baseline condition and during post-exercise hypotension (PEH).

	BASELINE	PEH
SBP, mmHg	115.6 ± 3.0	104.7 ± 3.0^*^
DBP, mmHg	70.0 ± 2.0	64.4 ± 2.1^*^
CO, L/min	4.2 ± 0.2	4.7 ± 0.2^*^
SV, ml	74.5 ± 2.6	82.1 ± 2.6^*^
HR, b/min	56.7 ± 2.0	57.4 ± 2.0
TPR, dynes sec^−1^ cm^−2^	1652.5 ± 71.7	1344.9 ± 44.7^*^
FVR, dynes sec^−1^ cm^−2^	138.0 ± 16.1	98.2 ± 10.5^*^
CVR, dynes sec^−1^ cm^−2^	48.3 ± 3.0	37.8 ± 2.6^*^

*Values are means ± SEM. SBP, systolic blood pressure; DBP, diastolic blood pressure; CO, cardiac output; SV, stroke volume; HR, heart rate; TPR, total peripheral resistance; FVR, forearm vascular resistance; CVR, calf vascular resistance. Hemodynamic data refer to a subgroup of patients (n = 15) exhibiting PEH*.

* *p < 0.05 vs. baseline*.

**Figure 4:**
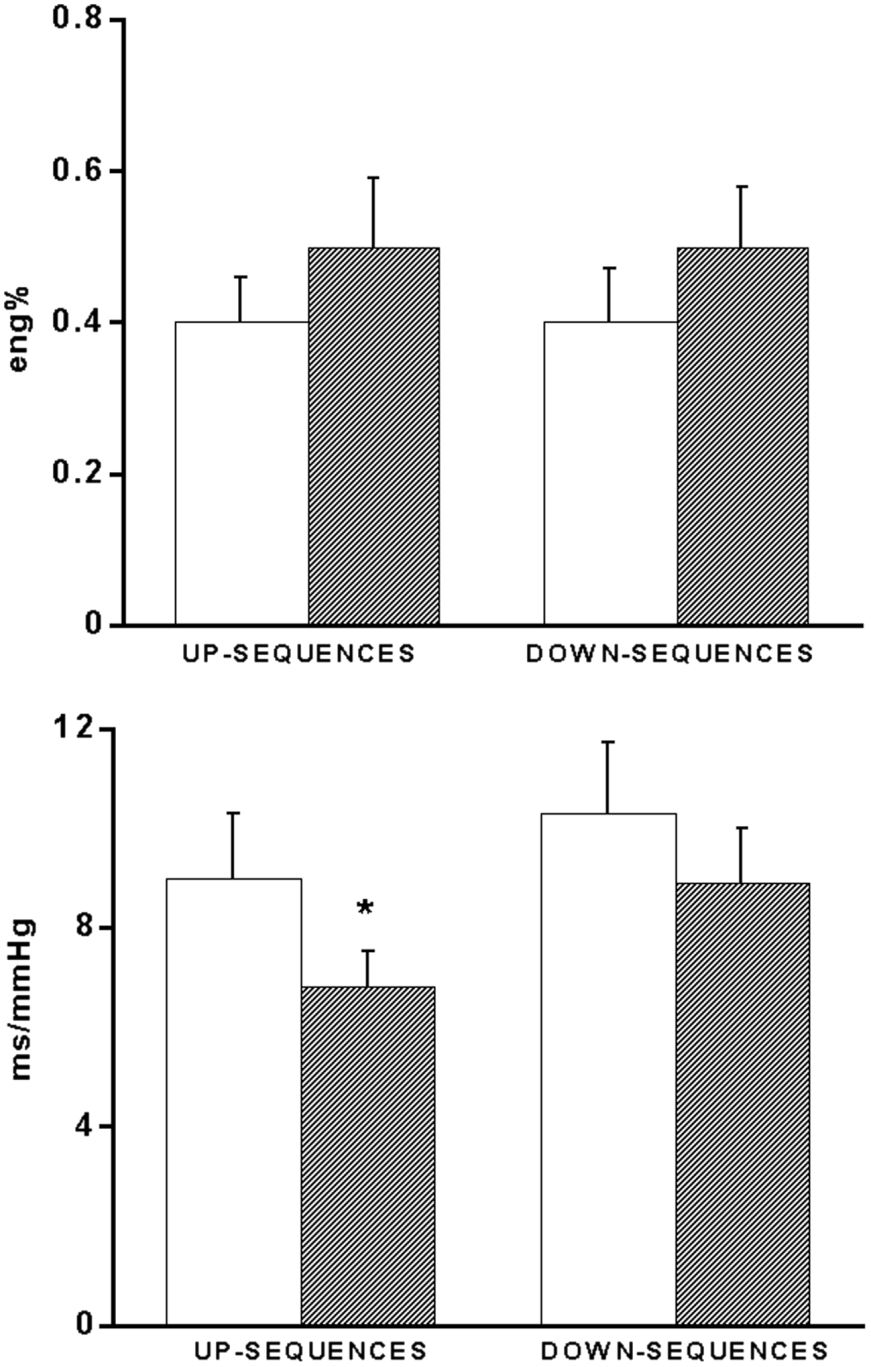
Bar graphs showing the occurrence (eng%, see methods) of up- and down-baroreflex sequences (upper panel) and baroreflex sensitivity values calculated separately for up- and down-baroreflex sequences (bottom panel) in 15 coronary artery patients baseline conditions (**open bars**) and 60 min after exercise (**hatched bars**). ^*^p < 0.05 vs. baseline.

In the same patients, BRS showed a significant (*p* < 0.05) decrease during supine recovery after exercise (7.5 ± 0.8 msec/mmHg) as compared to the pre-exercise values (9.8 ± 1.2 msec/mmHg). This decrease was mainly due to a significant BRS decrease in response to hypertensive stimuli (i.e., increasing BP ramps; Figure 4). No significant changes were observed for the occurrence of baroreflex sequences (Figure 4).

## Discussion

The novel finding of this study is that in CAD patients, the recovery period after exercise training session is characterized by a BP reduction similar to that usually observed in hypertensives. To our knowledge, this is the first study which addressed systematically BP behavior, with the attendant changes in central and peripheral hemodynamics, in the recovery phase after exercise in patients with CAD enrolled in an exercise training program.

PEH in our CAD patients differed from that observed in hypertensives in that BP showed a significant reduction only in systolic values, whereas a decrease both in systolic and in diastolic BP has been reported in hypertensives (Somers et al., 1991; Kenney and Seals, 1993; MacDonald et al., 2001; Legramante et al., 2002). The present study was not designed to define the mechanisms underlying PEH in different patients populations. Nonetheless, some observations deserve comments. Interestingly, the extent of systolic BP decrease after exercise in normotensive CAD patients was in a range similar to that reported for healthy subjects (Coats et al., 1989; Somers et al., 1991; Kenney and Seals, 1993). This observation suggests that the extent of BP decrease occurring in the recovery phase after exercise is linked to baseline BP levels. When BP is high, as in hypertensives, the cardiovascular regulatory mechanisms would allow a greater BP decrease after exercise as compared to normotensives (either healthy or CAD patients) in whom the regulatory system would act to protect from excessive BP decreases. We suggest that in CAD patients the arterial baroreflexes might play a key role in this protection (see below). The possibility that therapy (not discontinued in our patients) could have influenced the extent of BP reduction cannot be excluded. However, drugs were not withdrawn purposely for ethical reasons.

Similarly to previous reports in hypertensives (Pescatello et al., 1991; MacDonald et al., 2001), in our study, PEH was not limited to the early recovery but was sustained throughout a substantial part of the day. SBP showed a significant long-lasting reduction (~ 12 h) during the “exercise” day as compared to the “control” day. Moreover, even the nighttime BP reduction was significantly greater in the “exercise” as compared to the “control” day.

The sustained BP-lowering effect of exercise has potentially relevant clinical implications, adding to the other benefits of physical activity programs in CAD patients, even in those not suffering from concomitant hypertension history. In fact, the control of BP levels has been widely acknowledged as a major factor positively affecting the prognosis in CAD patients and uncontrolled SBP has been reported as an independent predictor of cardiovascular outcome in patients admitted to hospital for an acute coronary event (Amar et al., 2002). In this context, the possible role played by exercise as an add-on therapy in the control of systolic BP in CAD patients should be regarded as remarkable.

Our results differ from a previous study investigating a similar group of patients which showed no evidence of PEH (Fagard and Vanhees, 2000). Even though that study used an experimental protocol similar to ours, some differences deserve further comments. First, Fagard et al. (Fagard and Vanhees, 2000) measured BP shortly after the end of the exercise (~ 20 min) when patients had changed positions from supine to sitting. The well-known effects of body posture changes on BP regulation (Blomquist and Lowell-Stone, 1983) might have influenced the BP measurements. Second, the small sample size (*n* = 7) could have provided a partial representation of the post-exercise BP behavior in CAD patients. Finally, Fagard et al. (Fagard and Vanhees, 2000) studied patients participating in an outpatient cardiac rehabilitation program, whereas our patients resided in the Cardiac Rehabilitation Center, thus sharing the same daily routines and allowing us to carefully check their activities during the whole duration of the study.

### Central and Peripheral Hemodynamics

In keeping with most of the studies performed so far in hypertensives (Pescatello et al., 1991; Hara and Floras, 1995; Legramante et al., 2002), the present investigation confirms that also in CAD patients the decrease in TPR is the main mechanism sustaining PEH. However, at variance with hypertensives in which vasodilation would be limited to the previously active limb, that is, the lower legs (Legramante et al., 2002), in our CAD patients, the vasomotor responses appear similar in different regional vascular beds, being characterized by a significant and marked decrease in both CVR (i.e., the active limbs) and FVR (i.e., the non-active limbs), thus suggesting a generalized vasodilation. This latter was associated with an increase in SV, which may explain the observation of an increased CO despite an unchanged HR as compared to pre-exercise values. This finding suggests the indirect improvement in cardiac performance as an after-effect of a single exercise training session, at least in CAD patients with preserved left ventricular function, in line with previous observations in hypertensives (Cléroux et al., 1992; Hara and Floras, 1995; Legramante et al., 2002). This could also provide a temptative explanation of the improved left ventricular function reported in CAD patients undergone exercise training (Belardinelli et al., 1998).

### Spontaneous Baroreflex

A number of studies (Bennet et al., 1984; Somers et al., 1985; Hagberg et al., 1987; Convertino and Adams, 1991; Legramante et al., 2002) reported a lack of tachycardia during early PEH in hypertensives, raising the question of an alteration in arterial baroreflex modulation of HR. This prompted us to investigate whether this phenomenon is peculiar to hypertensives which have defective baroreflex control mechanisms (Pagani et al., 1988) or whether this phenomenon is generalized to other patients’ populations. Also in our CAD patients, BP decrease was not accompanied by the anticipated reflex tachycardia, and this was associated to a leftward shift in the baroreceptor-cardiac stimulus-response relationship to the lower BP level of post-exercise recovery, with a decreased overall BRS from pre-exercise value. This finding would suggest that the arterial baroreflex would be not simply reset (Pagani et al., 1988) along the prevailing (decreased) BP values of the post-exercise period [i.e., a pressure-dependent, acute “baroreceptor” resetting (Sagawa, 1983 Chapleau et al., 1989)], but would also actively contribute to maintain lower HR values through a decrease in baroreceptor-cardiac reflex gain, thus contributing to maintain low BP values by buffering the expected reflex tachycardia. However, BP decrease remained within safety level during recovery and our data suggest that the arterial baroreflex could contribute to this protective effect. In fact, the reduced BRS, due to the decrease in response to hypertensive stimuli (i.e., the increasing BP ramps), attempts to limit the bradycardia resulting from baroreceptors engagement on a recurring basis by spontaneous fluctuations in BP above the mean levels. This attenuated bradycardic pattern would contribute to prevent excessive BP decreases and could also exert a cardioprotective role in the setting of an acute decrease in BP after exercise, by avoiding an increase in myocardial oxygen consumption ensuing from a reflex tachycardia.

It thus appears that the lack of tachycardia during PEH is not limited to hypertensive patients but is a generalized phenomenon, which may indirectly contribute to the maintenance of post-exercise decrease in BP. Although more subtle differences in baroreflex regulation of sinus node may exist between different patients population (Legramante et al., 2002), overall, baroreflex control of HR seems to play substantially a permissive role in PEH also in CAD patients.

It is worth of note, however, that the arterial baroreflex appears capable of buffering excessive PEH by using different strategies. In fact, in hypertensives (Legramante et al., 2002) BRS has been shown to increase in response to *hypotensive* stimuli (i.e., decreasing BP ramps), whereas in the CAD patients of this study, BRS was decreased in response to *hypertensive* stimuli (i.e., increasing BP ramps). These findings suggest that the arterial baroreflex seeks the better strategy to maintain a homeostatic control of the cardiovascular system, and outline the plasticity of the neural networks.

### Limitations of the Study

One possible limitation of this study is the lack of a control group of non-CAD subjects. However, our interest was to test the hypothesis that PEH would occur in normotensive CAD patients and the mechanisms underlying PEH in this widely diffused patients’ population. Indeed, several studies have already demonstrated the occurrence of PEH in healthy subjects (in addition to hypertensives). Second, we did not evaluate CAD patients not featuring PEH, so we would not been able to ascertain whether baroreflex responses were returned to normal at 60 min, in line with BP recovery, This, however, would not detract from the novelty of the study. Third, stroke volume was measured by echocardiography, and this might have been a source of mistakes. However, our echocardiographers had a long experience; therefore, we are confident of our results. This study included only male CAD patients, and hence, our results cannot be generalized to females. Moreover, we employed only the aerobic continuous exercise modality. This choice was dictated by current guidelines that recommend aerobic exercise as the preferred training modality (Piepoli et al., 2016) Therefore, it is possible that different training modalities (e.g., resistance, combined, and high intensity interval training) could produce different results as far as PEH and its mechanisms is concerned (Iellamo et al., 2021). Finally, we cannot exclude the possibility that the prevalence of lower blood pressure values we observed might had been different if assessment had been performed at different times during the course of the training period. The last week of the training program was chosen because at that time, the patients had a lower likelihood to be deconditioned and this would have prevented from performing an effective training session.

## Data Availability Statement

The raw data supporting the conclusions of this article will be made available by the authors, without undue reservation.

## Ethics Statement

The studies involving human participants were reviewed and approved by Ethics Committee IRCCS San Raffaele Pisana, Roma. The patients/participants provided their written informed consent to participate in this study.

## Author Contributions

All authors listed have made a substantial, direct, and intellectual contribution to the work and approved it for publication.

## Conflict of Interest

The authors declare that the research was conducted in the absence of any commercial or financial relationships that could be construed as a potential conflict of interest.

## Publisher’s Note

All claims expressed in this article are solely those of the authors and do not necessarily represent those of their affiliated organizations, or those of the publisher, the editors and the reviewers. Any product that may be evaluated in this article, or claim that may be made by its manufacturer, is not guaranteed or endorsed by the publisher.
